# P-1814. Comparative Analysis of Receptor Binding Affinity and Potential Zoonotic Spillover of H5Nx Clade 2.3.4.4b Avian Influenza Virus from Bangladesh

**DOI:** 10.1093/ofid/ofaf695.1983

**Published:** 2026-01-11

**Authors:** Subyeta Binte Sarwar, Ayman Bin Abdul Mannan, Akash Saha, Md Hasibul Hassan, Mohammad Enayet Hossain, Sukanta Chowdhury, Mohammed Ziaur Rahman

**Affiliations:** icddr,b, Dhaka, Dhaka, Bangladesh; icddr,b, Dhaka, Dhaka, Bangladesh; icddr,b, Dhaka, Dhaka, Bangladesh; icddr,b, Dhaka, Dhaka, Bangladesh; icddr,b (International Centre for Diarrhoeal Disease Research, Bangladesh), Dhaka, Dhaka, Bangladesh; icddr,b, Dhaka, Dhaka, Bangladesh; icddr,b (International Centre for Diarrhoeal Disease Research, Bangladesh), Dhaka, Dhaka, Bangladesh

## Abstract

**Background:**

Highly pathogenic avian influenza H5Nx viruses of clade 2.3.4.4b have caused extensive outbreaks in wild birds and poultry worldwide, with sporadic human cases reported globally since 2022. Viruses of this clade possess the ability to breach the avian–mammalian species barrier. To date, no human cases have been reported in Bangladesh. Therefore, we investigated the mutational profile, receptor-binding affinity, and stability of circulating 2.3.4.4b avian strains in Bangladesh and compared them to 2.3.4.4b human strains isolated globally.

**Figure:**
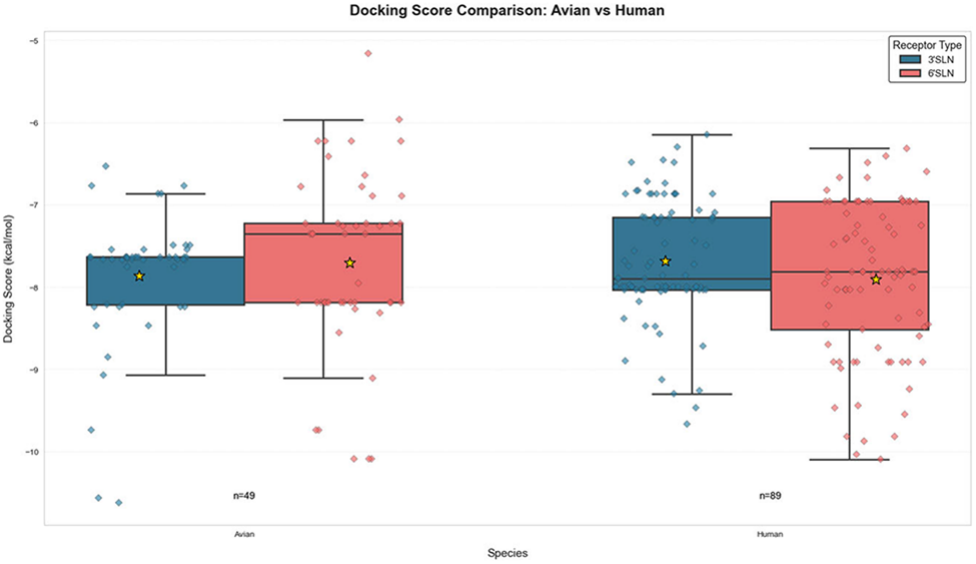
Molecular docking of avian influenza H5Nx strains of 2.3.4.4b clade with avian-type(3SLN) and Human-type receptors(6SLN) Molecular docking analysis of avian-and human- strains of 2.3.4.4b clade of influenza A H5Nx viruses with avian-type(3SLN) and Human-type receptors(6SLN). The star symbol represents the mean docking score.

**Methods:**

We retrieved complete sequences of clade 2.3.4.4b H5Nx avian strains (n=50, from Bangladesh) and human strains (n=89, worldwide) from GISAID and screened for mutational markers in the hemagglutinin (HA) segments. To assess the impact of these mutations, we conducted molecular docking of HA with avian and human receptor analogs—3′-sialylacetyllactosamine (3′-SLN) and 6′-sialylacetyllactosamine (6′-SLN)—using the GlideXP module in Maestro. We further analyzed dynamic stability through molecular dynamics simulations for up to 100 ns using the Desmond module in the Schrödinger suite.

**Results:**

All 2.3.4.4b viruses from Bangladesh had the polybasic cleavage site REKRRKRGLF. Despite the presence of several markers associated with increased binding to 6′-SLN in all strains, human-derived 2.3.4.4b strains exhibited additional adaptive mutations, including 104G (33%), 120M (40%), 131Q (39%), 172A (98%), 211I (37%), 226A (40%), 336N (29%), and 526V (39%). Docking analysis revealed that the mean binding affinities of avian 2.3.4.4b strains were –7.87 (95% CI: –7.64 to –8.09) for 3′-SLN and –7.71 (95% CI: –7.40 to –8.02) for 6′-SLN, whereas human 2.3.4.4b viruses showed stronger binding affinities of –8.2 (95% CI: –8.0 to –8.5) for 3′-SLN and –9.5 (95% CI: –8.8 to –10.3) for 6′-SLN.

**Conclusion:**

Our findings suggest that while Bangladeshi avian 2.3.4.4b viruses share important molecular markers with human strains and display moderate binding affinity toward human-like receptors, additional mutations—particularly 131Q and 172A found in human isolates—may enhance binding to human-type receptors, potentially facilitating zoonotic transmission and serious infections in humans.

**Disclosures:**

All Authors: No reported disclosures

